# Chronic Nonallergic Rhinosinusitis Associated With Military Burn Pit Exposure

**DOI:** 10.7759/cureus.84584

**Published:** 2025-05-21

**Authors:** Yoon Kyung Lee, Monica Tsai, Lorraine Anderson, Vivian Wang, Joseph Yusin

**Affiliations:** 1 Internal Medicine, UCLA Ronald Reagan Medical Center, Los Angeles, USA; 2 Allergy and Immunology, West Los Angeles VA Medical Center, Los Angeles, USA; 3 Clinical Immunology and Allergy, University of California Los Angeles, Los Angeles, USA

**Keywords:** burn pit exposure, chronic rhino-sinusitis, previously deployed, sino nasal disease, veteran’s health

## Abstract

Millions of previously deployed individuals nationwide have been affected by military toxic exposures and could be predisposed to a variety of associated health conditions. We present a case of a 38-year-old veteran with chronic rhinosinusitis with nasal polyps (CRSwNP) for two years and explore its possible association with burn pit exposure. He had a history of military burn pit exposure during his deployments to Iraq and Afghanistan, which spanned a total of 20 months. Physical examination revealed pale and enlarged inferior nasal turbinates. CT of the sinuses revealed pansinus mucosal thickening and polypoid tissue. Serologic testing for environmental allergies was negative. Flexible laryngoscopy revealed right nasal polyps. His presentation was consistent with a diagnosis of chronic nonallergic rhinosinusitis with nasal polyps. He was treated with fluticasone and ipratropium intranasal sprays, oral antihistamines, and saline sinus irrigations. Symptom control fluctuated during periods of nonadherence. He required a course of prednisone and intermittent courses of budesonide sinus irrigations. At the one-year follow-up, he had an excellent treatment response on intranasal sprays and intermittent budesonide sinus irrigations. Enhanced awareness of the impact of toxic exposures on previously deployed individuals’ health may help improve their quality of life.

## Introduction

Burn pits have been utilized during United States (US) overseas deployments, prominently in Iraq, Afghanistan, and parts of Southwest Asia, to dispose of military waste. This practice involves the burning of various materials, including chemicals, rubber, metals, and plastics, in open air, exposing the nearby surroundings to smoke, toxic fumes, and irritants. The impact of these toxins on veterans' health was recognized via the 2021 Veterans Burn Pits Exposure Recognition Act and the 2022 Promise to Address Comprehensive Toxics (PACT) Act, shedding light on the adverse health effects associated with deployment-related burn pit exposure. The presumptive health conditions following deployment-related burn pit exposure include upper and lower airway diseases, such as chronic bronchitis, chronic obstructive pulmonary disease (COPD), chronic rhinitis, and chronic sinusitis [1]. This report contributes to the existing body of literature by highlighting a case of chronic nonallergic rhinosinusitis with nasal polyps (CRSwNP) as a possible outcome for military burn pit exposure.

This report was previously presented as a poster at the 2025 American Academy of Allergy, Asthma, and Immunology (AAAAI) Annual Meeting on March 1, 2025.

## Case presentation

A 38-year-old male veteran was evaluated in the Allergy and Immunology clinic for symptoms of chronic rhinosinusitis for two years before presentation. In his 20s, he had experienced burn pit exposure during all his deployments: eight months in Camp Bucca, Iraq in 2007; six months in Afghanistan in 2008; and six months in Baghdad, Iraq in 2010. He stated that the “air was brown from dust or gray from burn pits, ash and debris in our sleeping quarters and everything … you want to exercise to pass the time, but you can’t because of the air.”

**Figure 1:**
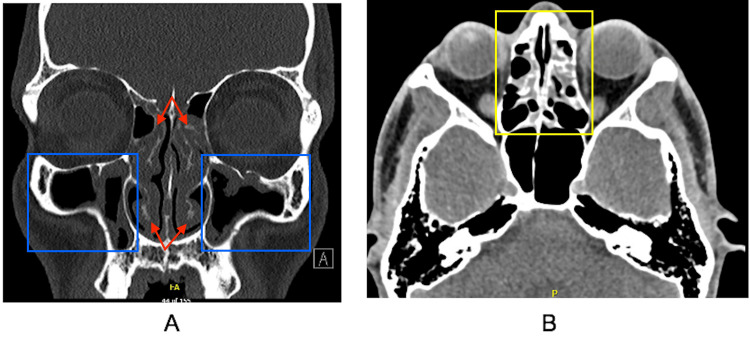
CT scan images of the sinuses Cross-sectional imaging revealing turbinate hypertrophy (red arrows) and polypoid changes of the (A) maxillary sinuses (blue boxes) and (B) ethmoid sinus mucosal thickening (yellow box) CT: computed tomography

His symptoms included rhinorrhea, post-nasal drip, nasal congestion that disrupted sleep, and sinus pressure that contributed to chronic headaches. He denied any history of asthma, hay fever, eczema, urticaria, stinging insect allergy, food allergy, or drug allergy. Physical exam showed pale, enlarged inferior nasal turbinates bilaterally with cobblestoning of the throat. CT of the sinuses showed pansinus mucosal thickening and polypoid tissue (Figure 1). Flexible laryngoscopy revealed a right septal polyp medial to the middle turbinate and another polyp more superiorly. Serologic immunoglobulin E environmental allergy testing was negative.

The patient's presentation was consistent with a diagnosis of CRSwNP. He was treated with nasal saline irrigations, fluticasone nasal spray, ipratropium nasal spray, and oral antihistamines. Symptoms fluctuated based on medication adherence. He required a course of prednisone and intermittent courses of budesonide sinus irrigations. He declined endoscopic sinus surgery. At the one-year follow-up, he had an excellent treatment response with physical exam showing reduction in turbinate hypertrophy and decreased pallor of the nasal mucosa. His symptoms were under control, without requiring sinus surgery.

## Discussion

In the first two years since the passing of the PACT Act, 5.7 million previously deployed individuals underwent toxic exposure screenings through the Veterans Affairs (VA) Health System [2]. Of note, 739,421 veterans were enrolled in VA healthcare, with more than 333,767 veterans enrolled from the PACT Act [2]. The Airborne Hazards and Open Burn Pit Registry (AHOBPR) was established in 2014 and encouraged further research to reveal how exposure to these toxins impacts veterans' health. Studies have sought to elucidate the causation between deployment-related burn pit exposure and airway disease.

Current literature supports a causative link between deployment-related burn pit exposure and the development of chronic rhinitis, chronic rhinosinusitis, and non-allergic rhinitis. In 2023, CHEST consensus statements on deployment-related respiratory disease emphasized that multiple exposures during deployment, including to burn pits, are associated with a variety of respiratory conditions, including chronic rhinitis and chronic rhinosinusitis [3]. A cross-sectional study of previously deployed individuals treated at a US Military Treatment Facility Rhinology clinic showed that deployment-related burn pit exposure is associated with increased sinonasal disease. This finding was supported by objective evidence of sinonasal disease as evidenced by nasal endoscopy and Sinonasal Outcome Test-22 scores showing that previously deployed individuals with burn pit exposure had significantly worse sinonasal disease outcomes as compared to those without exposure [4].

When comparing those with exposure to burn pits to those without, the rate of CRSwNP was determined to be significantly higher for the cohort with exposure [4]. Beyond sinonasal disease, one study revealed that in a cohort of AHOBPR veterans, deployment-related burn pit exposure was associated with increases in self-reported emphysema, chronic bronchitis, and chronic obstructive pulmonary disease (COPD) [5]. In 2024, a review of ICD coding in a cohort of nearly half a million veterans who served in Iraq and Afghanistan demonstrated that for every hundred days spent at bases with burn pits, previously deployed individuals had greater odds for airway diseases, including asthma and COPD [6]. However, additional research inquiry is needed to fully elucidate the mechanisms by which burn pit exposure may promote airway and sinonasal disease. More specifically, further research is needed to determine the pathophysiology of potential nasal polyp formation from burn pit exposure.

The pathophysiologic mechanisms of airway mucosal changes after deployment-related burn pit exposure are numerous and under investigation. The fumes from burn pits may contain toxins such as polycyclic aromatic hydrocarbons, volatile organic compounds, and heavy metals [7], which can result in short- and long-term health risks. Toxins in burn pit smoke may injure the airway mucosa, and there are studies demonstrating alterations in tissue function at the immunologic level. The impact of these toxins on sinonasal tissues overlaps with many of the mechanisms contributing to the pathogenesis of rhinosinusitis with nasal polyps, which includes mucosal epithelial barrier damage leading to enhancement of multiple types of inflammatory markers [8]. Higher levels of neutrophils, interleukin-6, and macrophage inflammatory protein-2 were identified after bronchoalveolar lavage of mouse models exposed to combustion of materials that may be found in military burn pits [7]. In addition, cultured human airway epithelial cells that were exposed to combustion of burn pit materials had increased cytotoxicity and upregulation of cytokines like interleukin-8, interleukin-15, and macrophage-derived chemokine (MDC) [9]. Although these inflammatory biomarkers were not measured for this case, they may serve as potential markers for future cases, potentially including trends of neutrophils and interleukins.

In recent years, there has been wider recognition of the impact of deployment-related burn pit exposure and the need to identify exposure for previously deployed individuals. Regulations on burn pits were implemented by the US Department of Defense in 2009 [10], and continued research on deployment-related burn pit exposure is warranted and supported by initiatives such as the Military Exposures Research Program from the US Office of Research and Development [11].

## Conclusions

As demonstrated in this report, chronic rhinosinusitis may be associated with deployment-related burn pit exposure and can impact previously deployed individuals’ well-being; standard-of-care treatments have the potential to improve their health. Enhanced awareness of the effects of toxic exposures on veterans’ health may aid in continuing to improve the quality of life of veterans. A systematic approach to evaluate sinonasal symptoms in previously deployed individuals may help appropriately identify and address post-deployment symptoms.
